# Use of SU8 as a stable and biocompatible adhesion layer for gold bioelectrodes

**DOI:** 10.1038/s41598-018-21755-6

**Published:** 2018-04-03

**Authors:** Bruno F. E. Matarèse, Paul L. C. Feyen, Aniello Falco, Fabio Benfenati, Paolo Lugli, John C. deMello

**Affiliations:** 1 Imperial College London, Exhibition Road, South Kensington, London, SW7 2AY UK; 2 Center for Synaptic Neuroscience and Technology, Istituto Italiano di Tecnologia, 16132 Genoa, Italy; 3 Faculty of Science and Technology, Free University of Bolzano - Bozen, 39100 Bolzano, Italy; 4 Department of Experimental Medicine, University of Genoa, 16132 Genoa, Italy

## Abstract

Gold is the most widely used electrode material for bioelectronic applications due to its high electrical conductivity, good chemical stability and proven biocompatibility. However, it adheres only weakly to widely used substrate materials such as glass and silicon oxide, typically requiring the use of a thin layer of chromium between the substrate and the metal to achieve adequate adhesion. Unfortunately, this approach can reduce biocompatibility relative to pure gold films due to the risk of the underlying layer of chromium becoming exposed. Here we report on an alternative adhesion layer for gold and other metals formed from a thin layer of the negative-tone photoresist SU-8, which we find to be significantly less cytotoxic than chromium, being broadly comparable to bare glass in terms of its biocompatibility. Various treatment protocols for SU-8 were investigated, with a view to attaining high transparency and good mechanical and biochemical stability. Thermal annealing to induce partial cross-linking of the SU-8 film prior to gold deposition, with further annealing after deposition to complete cross-linking, was found to yield the best electrode properties. The optimized glass/SU8-Au electrodes were highly transparent, resilient to delamination, stable in biological culture medium, and exhibited similar biocompatibility to glass.

## Introduction

The development of bioelectrodes that are transparent, biocompatible and capable of extended, stable operation in a broad range of biological media is a critical challenge for the field of bioelectronics^1–3^. Optoelectronic devices such as light-emitting diodes^4–8^, photodiodes^9–12^, and phototransistors^13^ have been deployed in multiple media, ranging from relatively mild interstitial fluids^14–19^ to corrosive solutions^20–22^. The most important properties for bioelectrodes are high mechanical and chemical stability, high conductivity, high transparency, and excellent biocompatibility. For this reason, thin-film (≪100 nm) gold is usually considered the electrode of choice for bioelectronic applications^23–25^. A further requirement of a bioelectrode, however, is strong adhesion to the surface on which it is deposited – often a transparent substrate material such as glass or quartz. Unfortunately, gold exhibits poor adhesion to both these materials, and typically requires the inclusion of a thin layer of an oxidative metal such as chromium to achieve adequate adhesion to the substrate^26^. Chromium, however, has significant disadvantages from a bioelectronic perspective, including cumbersome patterning procedures and potential cytotoxicity, leading to a strong demand for alternative adhesion layers. (Note, while some other metals such as titanium have been successfully employed as adhesion layers for conventional electronic applications, the cytotoxicity of these metals and their oxides is also an open question, see e.g. ref.^27^).

The commercial negative-tone photoresist SU8^28^ has previously been used as an alternative adhesion layer to chromium since it adheres well to multiple substrate materials and (once cross-linked) is chemically inert^29,30^. SU8 has the further advantage of being photo-patternable, allowing patterned electrodes with feature sizes down to a few microns to be readily fabricated using a simple two-step expose/develop procedure^31^. We have previously reported preliminary data^32,33^, showing SU8-supported gold electrodes to have favourable and moderately stable electro-optical characteristics in cell culturing medium, making them a potentially attractive choice for bioelectronics. Unfortunately, we found that, after prolonged periods of immersion in culture medium, gold deposited on SU8 had a tendency to develop wrinkles and cracks on the surface, reducing electrode durability and performance^32,33^. Here we show that the adhesion of Au to SU8 can be controlled by varying the relative amounts of thermal annealing before and after Au deposition, and report an optimized processing protocol that reduces the tendency for wrinkling and cracking. We further show that the viability of neurons grown on an exposed layer of SU8 exceeds that of neurons grown on chromium, implying that any localized loss of gold from an SU8/Au electrode should impair cell viability less than the equivalent loss from a Cr/Au electrode. Overall, the results suggest that SU8 can be used as an effective adhesion layer for gold bioelectrodes, offering levels of adhesion comparable to chromium, but with superior biocompatibility.

## Biocompatibility of adhesion layers

Biocompatibility is a prerequisite for applying any material in a bioelectronic or biomedical device. Previous reports^34–39^ have shown that, owing to its chemical inertness, SU8 does not adversely affect cell lines *in vitro*, which has led to its extensive use as an encapsulant for biomedical devices. The effects of evaporated elemental chromium on cell lines, by contrast, are largely unknown, although there has been considerable speculation about the potential cytotoxicity of Cr(III) and Cr(VI) ions^40–45^. While an adhesion layer should ideally remain physically isolated from the biological environment, there is always the risk that it may become partially exposed due to corrosion or physical cracking of the overlying metal layer. To evaluate the potential effects of such an exposure, we plated acutely prepared hippocampal neurons obtained from Sprague Dawley rats onto (i) bare glass, (ii) glass coated with 5 nm chromium metal, and (iii) glass coated with 1 μm fully cross-linked SU8-2 – a widely used SU8 formulation optimized for deposition of films <5 μm. Samples for neuronal use were incubated for 24 hr in culture medium at 37 °C, and again overnight in aqueous PLL [Poly-L-Lysine] coating solution at 37 °C. Samples were washed with deionized (DI) water prior to cell plating. The same protocol was employed to prepare glass, chromium, and SU8 samples. Neurons grown directly on glass, as well as on glass coated with the two adhesion layers, were then investigated using a fluorescence-based assay of viability (7, 14, 21, 28 Days *in vitro*; DIV) and electrophysiological characterization (14–18 DIV) as described below.

**Figure 1:**
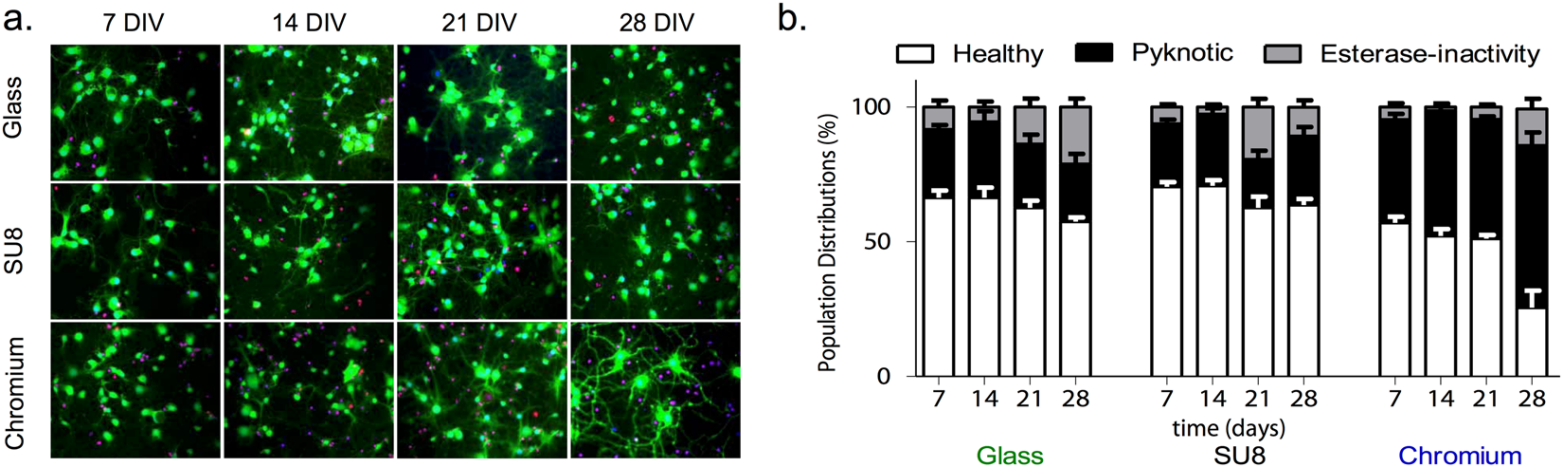
Viability of primary neurons on glass, cross-linked SU8-2 and chromium test-substrates. (**a**) Representative images acquired at one-week intervals, with green regions signifying viable cells, red/purple regions signifying pyknotic cells, and blue regions signifying cells without intracellular esterase activity (see Fig. S1 and Methods for details). (**b**) Stacked bar charts derived from fluorescence images, showing the distribution of viable, pyknotic and esterase-free cells versus time; error bars represent standard error of the mean. The plots show a significant influence of substrate material on cell viability (see main text), with the percentage of viable cells on chromium being significantly lower than on glass or SU8 at all time points, and the percentage of pyknotic cells being significantly higher.

To compare the population vitality of the hippocampal neurons on bare glass, Cr-coated glass, and SU8-coated glass, time-course experiments were carried out using two fluorescent probes (see Methods): the first probe – a fluorescein-based intracellular esterase activity-dependent reporter – was used to monitor cellular metabolism; while the second probe – a propidium iodide (PI) based DNA-intercalating probe (that cannot permeate healthy cellular membranes) – was used to monitor membrane integrity. Cells that were stained only with fluorescein were designated viable (healthy); cells that were stained with PI were designated pyknotic (i.e. having damaged chromatin in the nucleus^46^); and unstained cells were designated esterase-inactive (i.e. exhibiting dysfunctional cellular metabolism)^46^. For each substrate, data were obtained from 1280 ± 116 neurons at each time point, using images acquired from 3–4 replicate samples. Analysis was carried out by two-way ANOVA and Bonferroni post-test (p < 0.05). Figure 1 shows sample images at each time step, together with the quantification of viable, pyknotic and esterase-inactive populations versus time (see Methods and Fig. S1). The time course measurements revealed no significant differences between glass and SU8 in terms of the percentage of viable (healthy) cells at each time point. By contrast, the percentage of viable cells on Cr was found to be significantly lower at all time points. Non-viable cells on Cr were predominantly pyknotic. The higher percentage of pyknotic neurons measured at each time point on the Cr-coated substrate, indicates that the non-viable neurons on chromium were largely late apoptotic and necrotic^47,48^.

**Figure 2:**
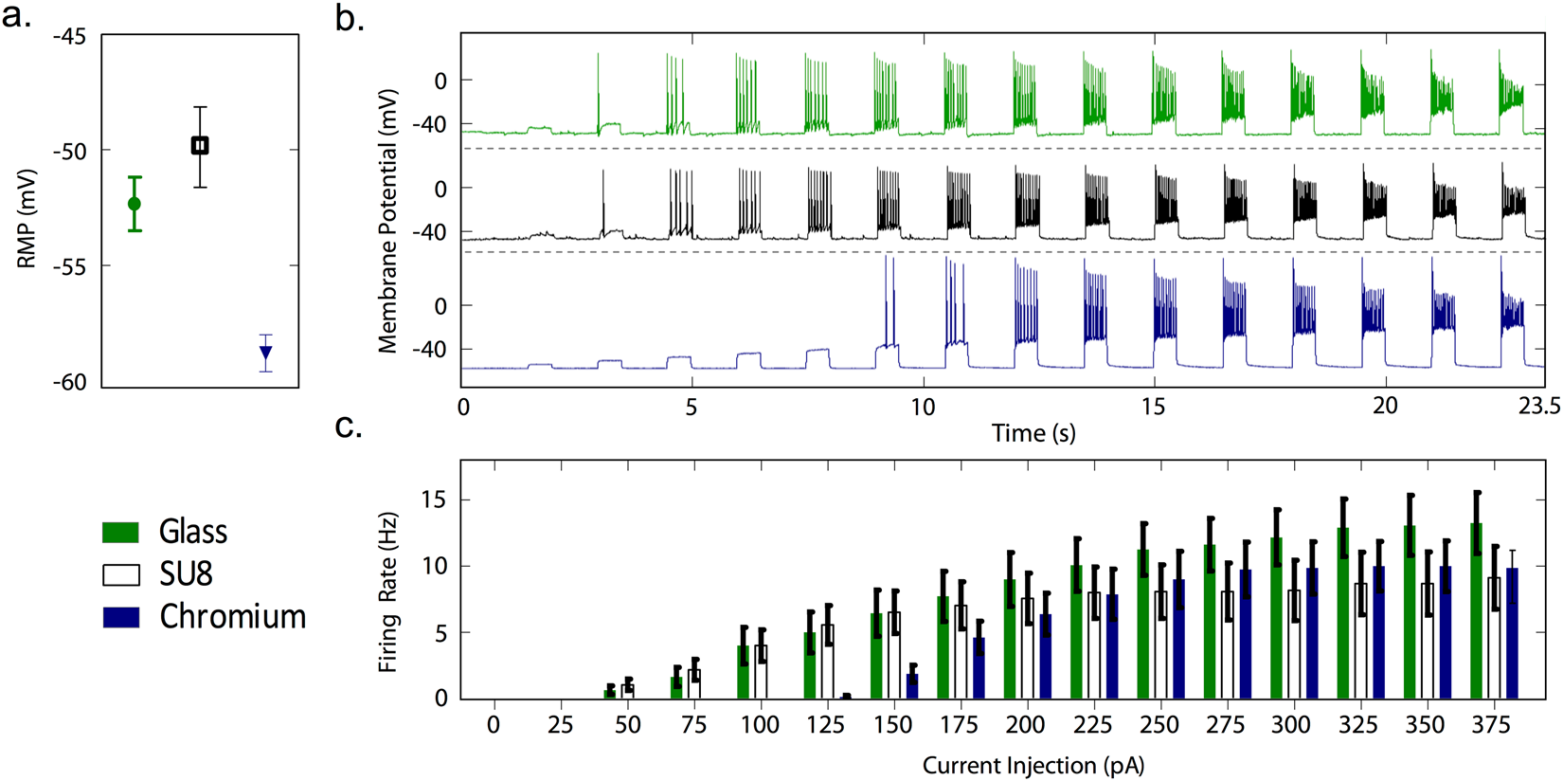
Electrophysiological characterization of primary neurons on glass, cross-linked SU8-2 and chromium test-substrates. (**a**) Plot of mean resting membrane potential (RMP, *ϕ*) versus substrate material, indicating a significantly higher mean RMP for neurons grown on chromium (*ϕ* = −58.79 ± 2.99 mV, *N* = 14) than either glass (*ϕ* = −52.33 ± 5.3 mV, *N* = 21) or SU82 (*ϕ* = −49.81 ± 7.81 mV, *N* = 21). Statistical analysis was carried out using two-way ANOVA followed by Bonferroni’s multiple comparison test. (**b**) Representative traces of elicited action potential versus time, recorded under current clamp conditions using a sequence of 500 ms current pulses, increasing from 0 to 375 pA with a step-size of 25 pA. (**c**) Bar charts showing for each substrate material the mean firing rate versus current pulse amplitude, indicating a higher action potential firing threshold for neurons grown on chromium but no significant differences in the firing rates between substrates.

Patch-clamp recordings^49^ were subsequently used to investigate the physiological state of hippocampal neurons on the three surfaces. The phenotype of a neuron is largely defined in terms of its ability to generate action potentials, the principal mechanism for rapid intra-neuronal data transmission and the initiator of inter-neuronal signaling at synapses^47,48^. We first evaluated the functional state of the neurons by quantifying their resting trans-membrane potentials. (Fig. 2a) Neurons grown on chromium showed hyperpolarized resting membrane potentials compared to those grown on glass (meaning an increased stimulus was required to move the membrane potential to the action potential threshold)^50–52^, while those grown on SU8 had similar resting membrane potentials to those grown on glass. We subsequently investigated the response of the neurons to electrical stimulation (Fig. 2b), using a current step protocol in which neurons were subjected to a train of 500 ms current pulses of progressively increasing amplitude (0–375 pA, step-size 25 pA, see Methods). At higher levels of current injection, neurons on bare glass, Cr-coated glass and SU8-coated glass all showed similar firing rate plateaus in the range of 8 to 13 Hz, with significance testing indicating no significant variations in firing rate at different current steps or between samples. However, neurons grown on Cr-coated glass showed significantly higher action potential firing thresholds (~150 pA) relative to those grown on bare glass and SU8 (~50 pA).

While it cannot be determined from these measurements if the alterations in neuronal behavior observed with chromium are directly caused by chromium or if they are a secondary effect of the increased cell death in the neuronal network, the findings indicate strong alterations in the functional properties of the neurons grown on the metallic adhesion layer. Similar findings of chromium toxicity were recently reported^41^ in orthopedic implant patients, where chromium-induced oxidative damage was posited to cause necrosis in the peri-implant tissue. *In vitro* studies of Cr_2_O_3_ nanoparticles^53^ have further shown chromium particulate toxicity to derived cell lines. The striking reductions in cell viability and the altered electrophysiological character of neurons in contact with evaporated chromium films strongly motivate the use of alternative adhesion layers in bioelectronic and medical applications. Given the similar behavior of neurons on bare glass and SU8-coated glass, SU8 appears to be a promising candidate for such an adhesion layer, offering excellent biocompatibility even with sensitive brain tissue cultures.

## Processing of SU8 adhesion layers

**Figure 3:**
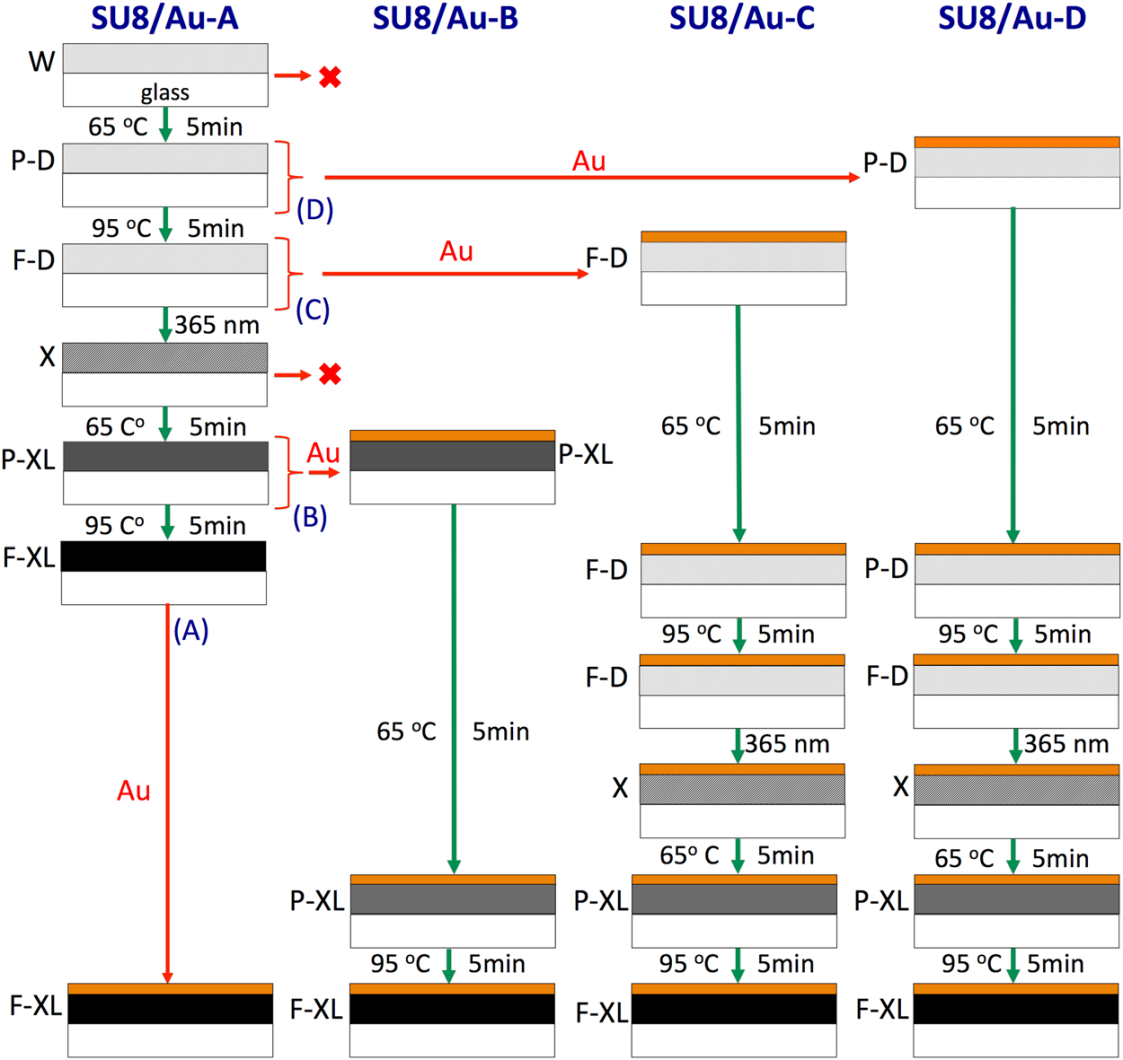
Schematic showing conventional protocol for preparing Au-coated SU8 on glass plus several variants employed in this work. In the conventional procedure (**A**) SU8 is first deposited onto a glass substrate from solution, typically by spin-coating; the film is dried and solidified by heating to 65 °C, and then to 95 °C; it is then cooled to room temperature (RT) and exposed using 350–400 nm UV radiation to initiate photo-acid generation; it is then heated again to 65 °C, and then to 95 °C to induce complete cross-linking; and finally it is cooled to room temperature for metal deposition under vacuum. In the conventional approach (**A**), Au deposition occurs onto fully cross-linked SU8 at the final stage directly after the second 95 °C heating stage. In the variant methods, Au deposition occurs onto partially cross-linked SU8 at earlier stages in the process, either after the first 65 °C heating stage (**D**), after the first 95 °C heating stage (**C**), or after the second 65 °C heating stage (**B**). In cases (**B**) and (**D**), where gold deposition takes place after a 65 °C heating stage, an additional 65 °C heating stage is added prior to the 95 °C stage to avoid cracking the film. Uncoated SU8 films were prepared by carrying out the procedures up to (but not including) the point of gold deposition. The condition of the film at each stage of processing is indicated by the black lettering: “W” denotes a wet film containing significant residual solvent; “P-D” denotes a partially dried film containing some residual solvent; “F-D” denotes a fully dried film, containing minimal residual solvent; “X” denotes a UV-exposed film; “P-XL” denotes a partially cross-linked film; and “F-XL” denotes a full cross-linked film.

SU8 comprises a mixture of an epoxy-based monomer, a solvent, and a photoacid initiator^28^. Under UV illumination the photoacid protonates epoxy moieties, enabling them to thermally react with any remaining neutral epoxy groups to yield a cross-linked polymer network. By varying the UV dose and curing time, the number of cross-linking sites can be controlled, and the mechanical stability of the film can therefore be modified^28,54–58^. When using SU8 conventionally (i.e. as a photoresist), the SU8 film is normally subjected to an initial (soft) bake to drive off excess solvent and solidify the film, reducing the risk of subsequent swelling and adhesion failure. The soft-bake is typically carried out in a three-step process to reduce stress, with the film first being warmed to ~65 °C, then being held at this temperature for a few minutes to allow the polymer chains time to relax and realign, and finally being ramped to 95 °C to drive off the excess solvent. The film is then allowed to cool slowly to room temperature, ready for UV exposure through an inverse photo-mask. Following exposure, the SU-8 is subjected to a post-exposure bake (PEB) to complete the cross-linking, often using the same 65/95 °C heating procedure. The overall procedure is summarized in Fig. 3 (see process labelled SU8/Au-A).

When using SU8 as an adhesion layer for gold bioelectrodes, it is not clear whether the standard processing protocol is the most appropriate for achieving optimal stability of the SU8/Au electrode in biological media. In particular, it is not obvious at which stage curing and UV exposure of the SU8 should best be carried out – before or after gold deposition? Figure 3 shows that there are six possible stages at which the Au deposition can be carried out. Two of these stages (denoted by ✖’s in the figure), however, may be excluded on practical grounds: evaporation of Au onto as-deposited ‘wet’ SU8 gives poor quality films due to permeation of Au into the polymer resin, while evaporation of Au immediately after UV exposure gives inconsistent results due to the difficulty of ensuring consistent diffusion of the photo-acid at the point of gold deposition. Four sets of SU8/Au films based on Au deposition at the remaining four stages (denoted by A, B, C and D in Fig. 3) were accordingly prepared: set A was prepared by carrying out the usual full protocol described above; set B was prepared by following the standard protocol up to and including the exposure step, followed by annealing at 65 °C to enable photoacid diffusion, Au deposition, and the remaining hard bake; set C was prepared by first carrying out a soft-bake, then depositing Au, and then carrying out the full standard procedure (ending after the hard bake); finally, set D was prepared by annealing at 65 °C prior to Au deposition, followed by the full standard procedure (ending after the hard bake). All annealing times were set to five minutes. The UV dosage for the exposure step was set to 100 mJ/cm^2^, a typical value for sub-micron film thicknesses. In passing from set A to set D, the degree of cross-linking at the time of metal deposition progressively decreased, meaning that Au was deposited onto a hard fully cross-linked film for set A, while it was deposited onto a tacky non cross-linked film for set D. The effects of the different processing protocols on the physical properties of the resulting bio-electrodes were investigated in a series of comparative studies described below.

## Stability of SU8 and SU8/Au films in culturing medium

*In vitro* bioelectronic investigations are typically carried out in cell culturing media^59–61^ that contain essential ions, peptides, lipids, enzymes, growth factors and other additives and products of metabolism of the cell lines under investigation. Despite the typically corrosive nature of such media^62–64^, it is often necessary for bioelectrodes to remain operational over many days or weeks, e.g. to monitor the cell state over physiologically relevant periods of time. To determine the suitability of electrodes A to D for use in culturing media, two sets of experiments were undertaken: the first one using bare SU8 films prepared according to the four protocols described above up to (but *not* including) the metal deposition, and the second one using Au-coated SU8 prepared according to the full protocols.

**Figure 4:**
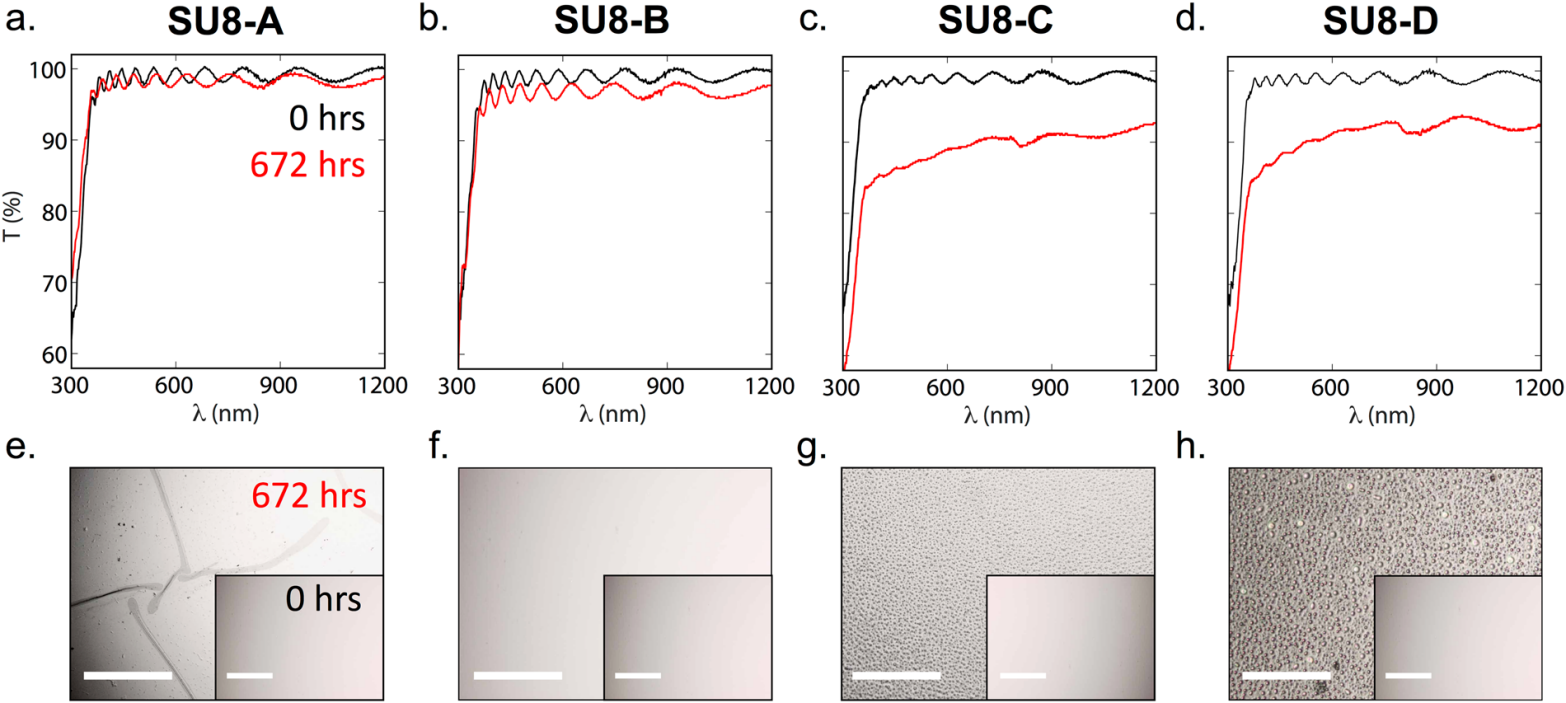
Optical characterization of SU8 films before (*t* = 0) and after (*t* = 672 hr) immersion in cell culture medium. Films were prepared by following protocols A to D in Fig. 3 up to (but not including) the step of Au deposition: (**a**–**d**) plots of transmittance versus wavelength at *t* = 0 and *t* = 672 hr, showing slight changes for SU8-A and SU8-B and much stronger changes for SU8-C and SU8-D after immersion; (**e**–**h**) micrograph images acquired at *t* = 0 hr (inset) and *t* = 672 hr (main images), showing cracks in SU8-A, wrinkles in SU8-C and SU8-D, and a largely intact film for SU8-B after immersion. Scale bars denote 50 μm.

For the first set of measurements, SU8-coated glass substrates of types A to D (labeled SU8-A, SU8-B, SU8-C and SU8-D) were immersed in cell culture medium [Dulbecco’s Modified Eagles Medium (DMEM)] and removed intermittently for analysis by transmission spectroscopy (Fig. 4a–d) and optical microscopy (Fig. 4e–h). Initially (0 hrs), all samples appeared uniform under an optical microscope and exhibited broadly similar transmission spectra with transmittances of >96% in the wavelength range 400–1200 nm. However, clear differences in both the optical micrographs and the transmission spectra emerged with time. After 672 hours of immersion, the most cross-linked sample SU8-A developed sizeable cracks. Over the same period of immersion, the next most cross-linked sample SU8-B remained optically clear, while samples SU8-C and SU8-D both developed a cloudy, granular appearance with the grain size being largest in the case of the least cross-linked sample SU8-D. While the transmission spectra of the two most cross-linked samples (SU8-A and SU8-B) were broadly unaffected by immersion in the culture medium, the other two samples showed a substantial (~10%) drop in transmission due to scattering by sub-micron sized features. From these measurements, it is evident that deposition of gold onto a fully cross-linked sample of SU8 may cause later damage to the gold layer due to fracturing of the underlying SU8. At the same time, insufficient cross-linking of the SU8 results in an unstable film that delaminates from the underlying substrate, with a commensurate loss in transparency due to scattering. The above findings are consistent with previous investigations into the swelling, and subsequent deformation, of SU8 under exposure to solvents and solvent vapours, see e.g. ref.^65^. Deformation has been attributed to spatial variations in the cross-linking density, which in turn lead to spatial variations in the amount of swelling – and hence strain – within the film: strongly cross-linked regions undergo only slight swelling, leading to low strain, while weakly cross-linked regions undergo strong swelling, leading to high strain. The fracturing of the fully cross-linked sample SU8-A is consistent with that sample undergoing very non-uniform swelling under the described processing conditions.

**Figure 5:**
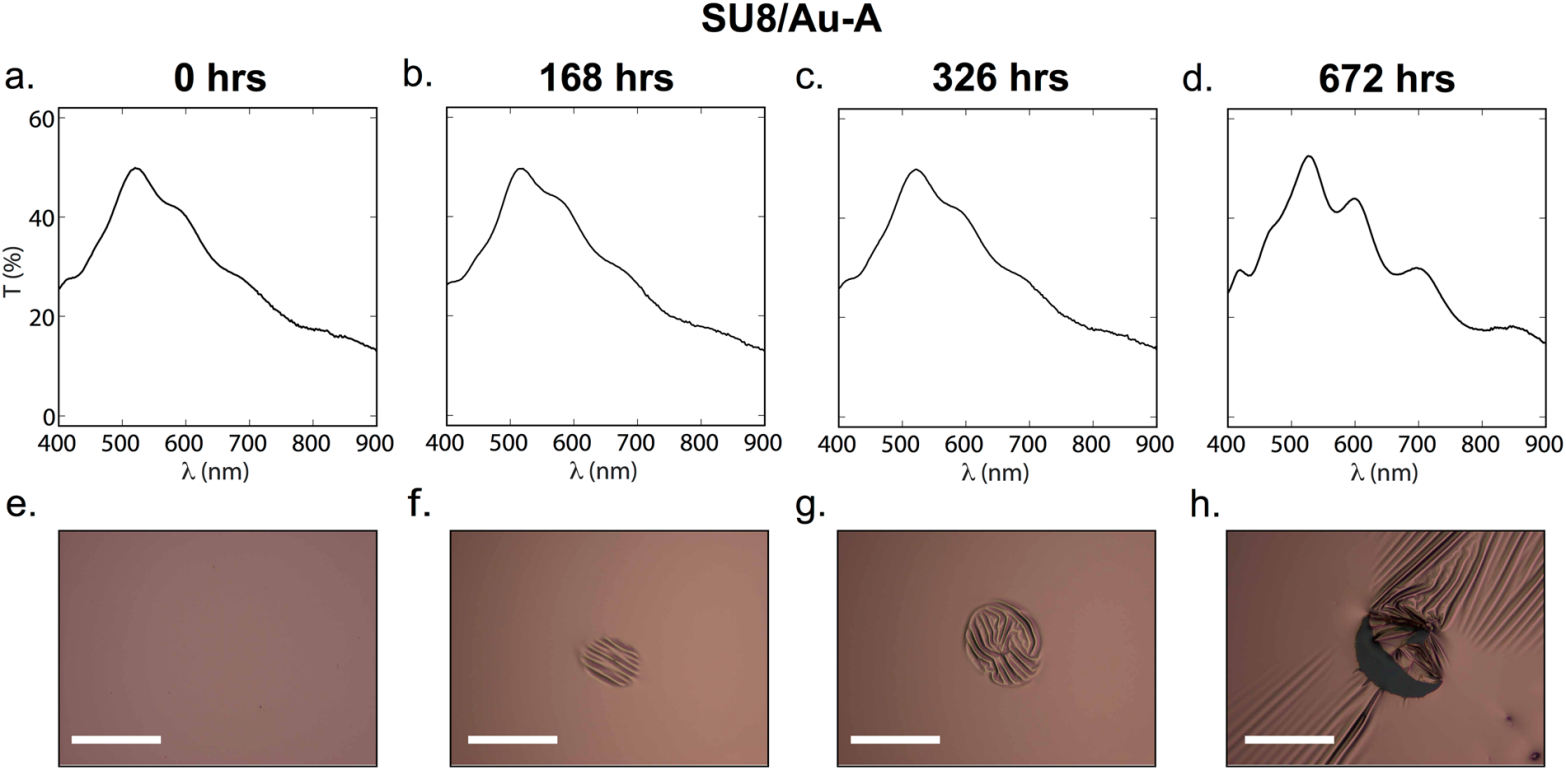
Evolution of optical characteristics of SU8/Au-A films with immersion time (the films were prepared by following Protocol A in Fig. 3). (**a**–**d**) Plots of transmittance versus wavelength at *t* = 0, 168, 326 and 672 hr, showing good stability in the spectral characteristics up to 326 hrs, followed by significant changes thereafter. (**e**–**h**) Reflection micrographs at *t* = 0, 168, 326 and 672 hr, showing wrinkling (168, 326 hr) and eventual tearing of the SU8/gold bilayer (672 hr). Scale bars denote 50 μm.

Previous reports^32,33^ involving the use of SU8 as an adhesion layer have typically entailed full processing of the SU8 film *before* the metal layer is deposited in accordance with protocol A. Figure 5a–d shows a series of transmission spectra recorded at 168-hour intervals for a sample prepared in this way (SU8/Au-A). The initial transmission spectrum was broadly characteristic of the transmission spectrum of thin-film gold, with some additional oscillatory structure due to the weak cavity formed by the glass/SU8/Au structure. Over time, a slight increase in peak transmittance was observed, together with the emergence of stronger oscillatory features. The observed behavior is consistent with the localized removal of SU8/Au and/or Au from the three-layer stack, leading to a combination of the initial SU8/Au-A spectrum and strongly oscillatory features due to SU8-A and/or SU8/Au-A. This behavior can be seen in the corresponding reflectance (Fig. 5e–h micrographs for sample SU8/Au-A: the formation of diagonal and parallel folds in the stack is evident at *t* = 168 hours, implying weak bonding of SU8 to the glass and/or weak bonding of Au to the fully cross-linked SU8 at this location; by *t* = 336 hours, stress due to immersion in the culture medium had distorted these folds into a vortex-like pattern; while, by *t* = 672 hours, complete detachment had occurred.

**Figure 6:**
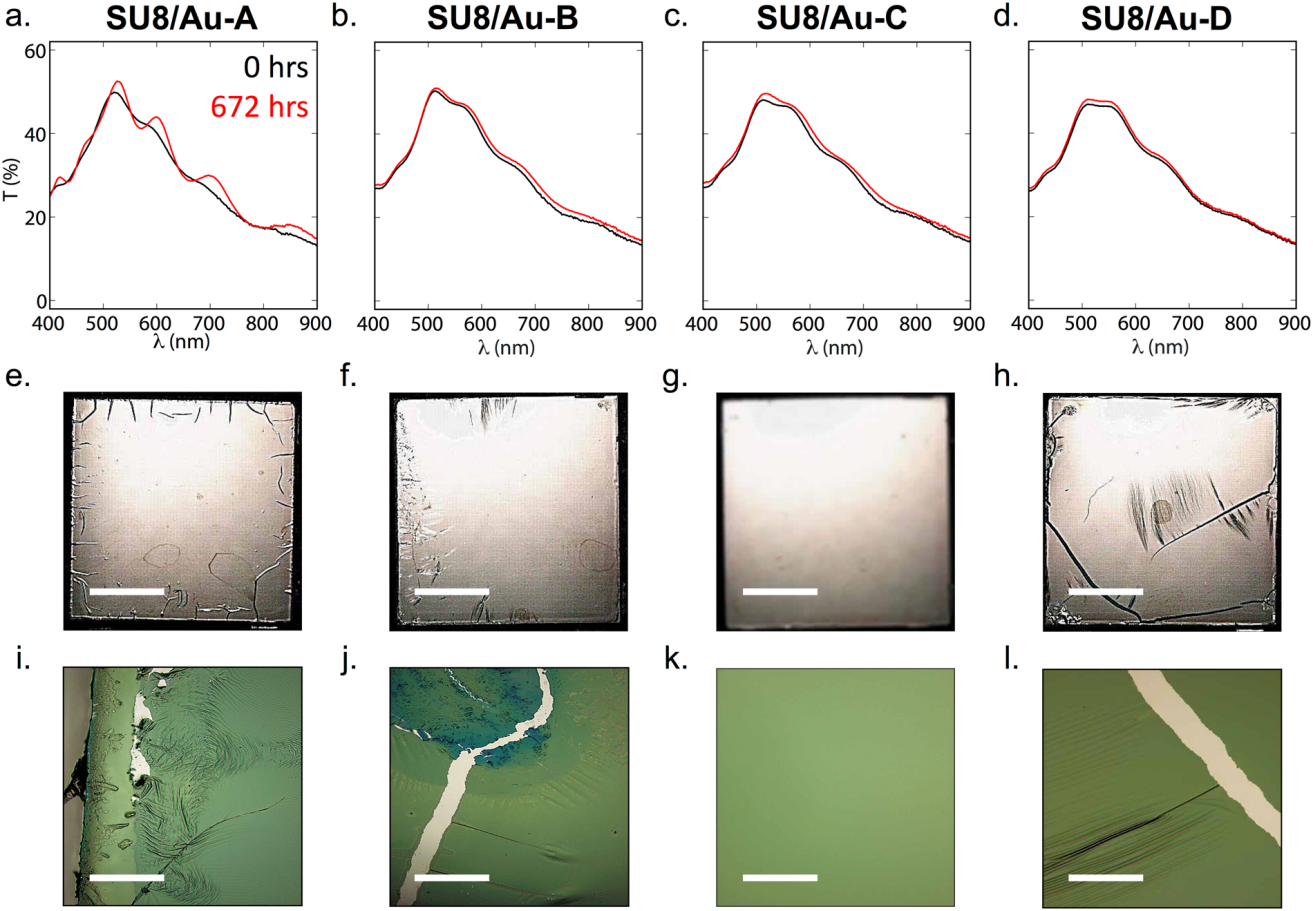
Optical characterization of SU8/Au films prepared by following protocols A to D in Fig. 3: (**a**–**d**) plots of transmittance versus wavelength before (*t* = 0 hr) and after (*t* = 672 hr) immersion in cell culture medium, showing substantial changes for SU8/Au-A but comparatively small changes for the other films; (**e**–**h**) micrograph images of entire samples before and after immersion, showing significant cracking after immersion in all films except SU8/Au-C. Scale bars denote 10 mm. (**i**–**l**) micrograph images before and after immersion, showing significant cracking after immersion in all films except SU8/Au-C. Scale bars denote 50 μm.

The SU8/Au samples prepared using protocols B, C and D (labeled SU8/Au-B, SU8/Au-C and SU8/Au-D) exhibited initial transmission spectra similar to SU8/Au-A (see Fig. 6a–d). However, they showed much weaker spectral changes with time, suggesting smaller changes in the morphology of the stack. However, comparing the reflectance (Fig. 6e–h) and transmittance (Fig. 6i–l) micrographs of the four samples obtained at *t* = 672 hours, it was evident that SU8/Au-B and SU8/Au-D had undergone qualitatively similar levels of degradation to SU8/Au-A, with both samples showing regions of wrinkling, cracking and delamination. Strikingly, sample SU8/Au-C remained optically uniform with no signs of film damage. Hence, taking the results from Figs 2, 3 and 4 together, it is clear that substantial cross-linking of SU8 is required to achieve strong adhesion of SU8 to glass, while Au should be deposited before cross-linking of the SU8 is complete to achieve strong adhesion of SU8 to Au.

**Figure 7:**
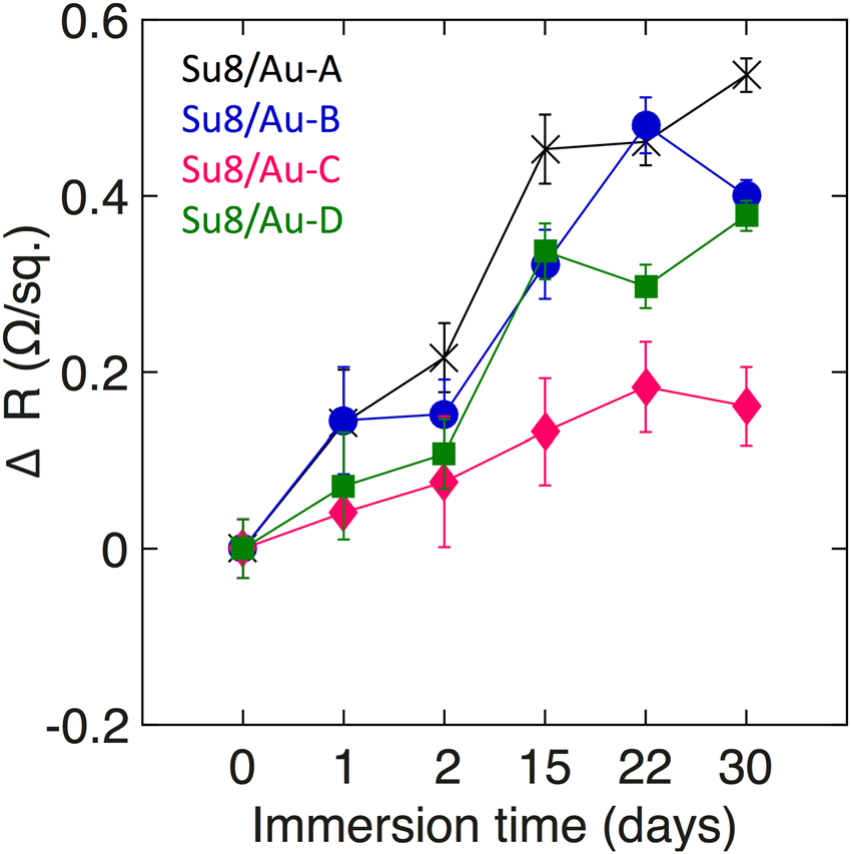
Plot of sheet resistance versus immersion time in cell culture medium for SU8/Au films prepared according to protocols A to D in Fig. 3.

Changes in the electrode performance as a result of prolonged immersion in the cell culture medium were assessed by carrying out four-point probe measurements of sheet resistance (see Methods) before immersion and after one day, two days, one week, two weeks and one month of immersion. Figure 7 shows plots of the change in sheet resistance versus immersion time for the four SU8/Au samples. All four samples showed a progressive increase in sheet resistance with the immersion time from a similar starting point of ~4.8 Ω/sq. before immersion. However, the SU8/Au-C sample showed a significantly slower rate of degradation, increasing by a small amount to ~4.9 Ω/sq. after one month (compared to ~5.2 Ω/sq. for the other samples), consistent with the higher stability of the SU8/Au-C electrode evident from the micrographs of Fig. 6g,k.

**Figure 8:**
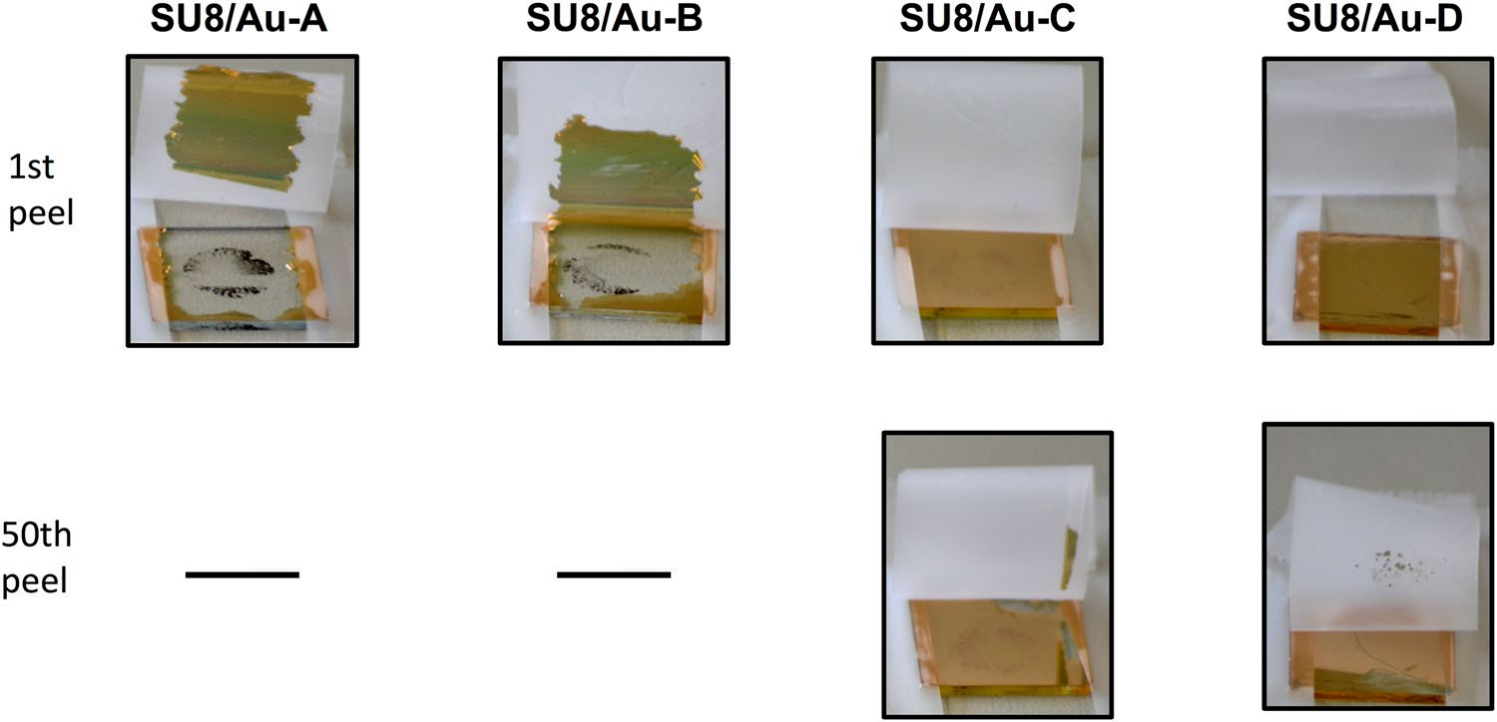
Images of SU8/Au films prepared according to protocols A to D in Fig. 3 after one peeling step (1^st^ row) and fifty peeling steps (2^nd^ row) using adhesive tape. In the case of SU8/Au-A and SU8/Au-B, images are shown only for the first peeling step due to immediate damage to the films.

The resilience of the SU8/Au films to mechanical disturbances was investigated using qualitative peel testing (see Fig. 8 and Methods). A strip of adhesive tape was applied to the gold surface and then peeled from the gold film at a constant speed of 50 mm/s at an angle of 90° to the surface. The process was repeated fifty times or until delamination of the gold film occurred. Samples SU8/Au-A and SU8/Au-B – i.e. the two films in which the SU8 was most heavily cross-linked at the time of gold deposition – suffered significant delamination within a single peeling step, indicating that Au does not adhere strongly to heavily cross-linked SU8. Samples SU8/Au-C and SU8/Au-D, by contrast, showed good resilience to repeated peeling steps and remained largely intact even after fifty repetitions. The improved adhesion of gold to SU8 in circumstances where cross-linking is initiated *after* gold deposition has occurred was recently investigated by Merschrod and co-workers^29^. Their studies indicate that, during post-deposition cross-linking, the SU-8 chains cross-link around the bottom layer of the gold atoms. This cross-linking and the subsequent shrinkage of polymerized SU-8 (due to the reduction in free volume) trap the bottom layer of gold atoms, resulting in a gold-polymer composite structure with good adhesion of the gold layer to the cured SU-8.

On the basis of the peel tests, it is evident that the SU8 should be only weakly cross-linked prior to gold deposition to ensure good adhesion between the two layers. It is further evident from the images of Fig. 6b that sample SU8/Au-C underwent the smallest amount of cracking and wrinkling when immersed in culture medium. Hence, on the basis of the data presented above, protocol C appears to be the most promising for the realization of stable bioelectrodes on glass substrates.

## Biocompatibility of SU8/Au layers

**Figure 9:**
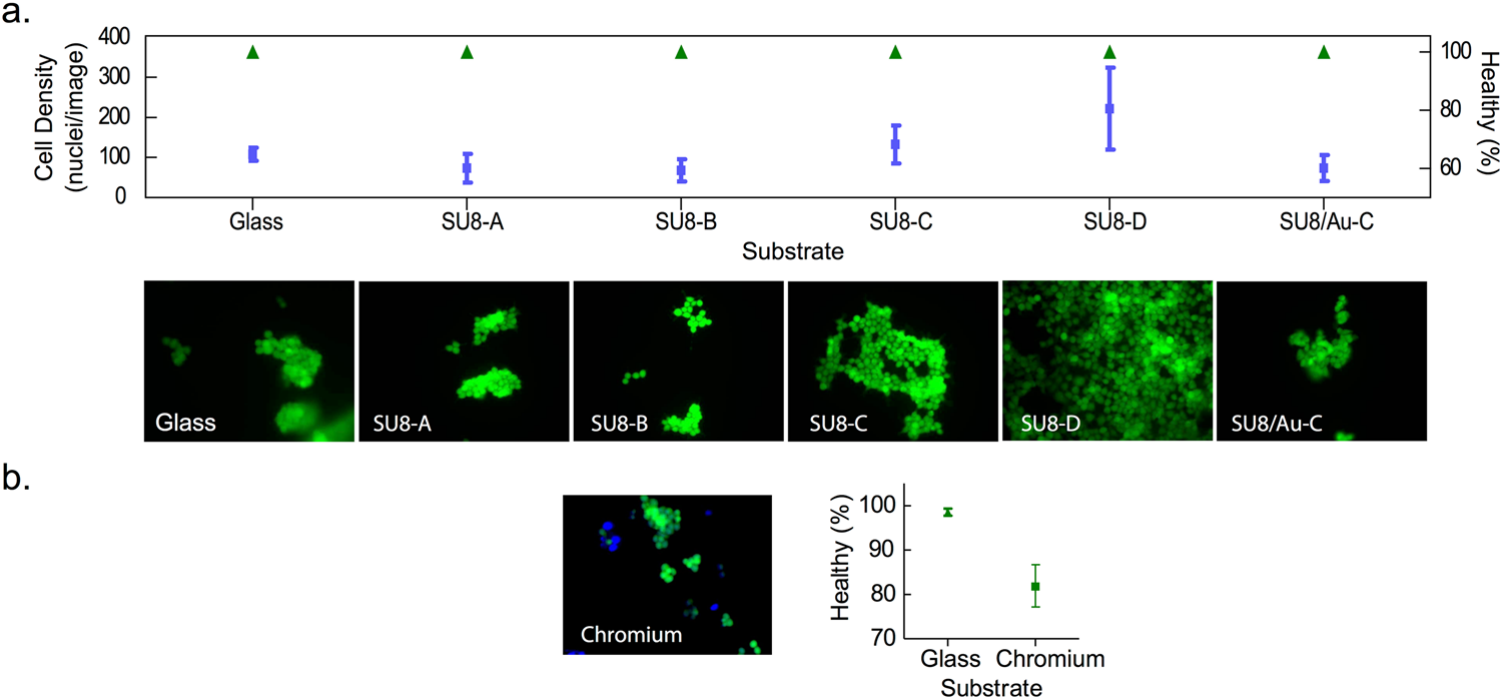
HEK293-T cell viability on glass, SU8 films prepared according to protocols A to D in Fig. 3, Cr and Au-coated SU8 prepared according to protocol C in Fig. 3. (**a**) Fluorescence micrographs of HEK293-T cells plated on samples of SU8-A-D, and SU8/Au-C, indicating no significant differences in cell adhesion (squares) or cell viability count (triangles) with respect to glass. (**b**) Fluorescence micrographs of HEK293-T plated on chromium, showing a significant reduction in viable cell population with respect to glass.

To assess the effect of the various processing protocols on SU8 biocompatibility, we compared HEK293-T cell adhesion and viability on bare glass, Cr-coated glass, glass coated with the differentially cross-linked SU8 samples (SU8-A-D) and glass coated with the optimized SU8/Au bilayer (SU8/Au-C). The samples were sterilized in 70% ethanol, prior to overnight incubation by 0.1% PLL solution. Adhesion was quantified using the Hoechst DNA stain, and cell vitality was assessed by FDA staining. All samples exhibited a similar level of cell adhesion, although the mean level of cell adhesion increased marginally from SU8-A to SU8-D. There were no significant differences in cell viability between bare glass, SU8-coated glass samples and SU8/Au-coated glass samples. However, for the Cr-coated glass sample, there was a significant (~20%) reduction in viable cell population relative to glass controls (*P* < 0.005, Mann-Whitney, one tailed), providing further evidence of its cytotoxicity and the superiority of SU8 as an adhesion layer for bioelectrode applications (Fig. 9).

## Discussion and Conclusion

In conclusion, we have described an effective protocol for fabricating mechanically and chemically stable gold bioelectrodes on glass, using a 1 μm layer of the negative-tone photoresist SU8 as an adhesion layer between the glass substrate and a thin layer of gold. Using a fluorescence-based assay of neuronal cell viability and electro-physiological characterization, SU8 was found to exhibit superior biocompatibility to chromium – the most widely used adhesion layer for gold on glass. Compared to neurons grown directly on glass, neurons grown on chromium-coated glass exhibited increased levels of cellular membrane permeability, consistent with elevated levels of apoptotic/necrotic processes; they also showed significantly higher action potential firing thresholds, suggesting an impairment of the neuronal phenotype. Neurons grown on SU8-coated glass, by contrast, showed similar behaviour to those grown directly on glass. Various treatment protocols for the SU-8 were investigated, with a view to attaining high transparency and good mechanical and biochemical stability. Thermal annealing to induce partial cross-linking of the SU-8 film prior to gold deposition, with further annealing after deposition to complete cross-linking, was found to yield the optimum electrode properties. From a combination of optical microscopy, transmission spectroscopy and four-point probe sheet resistance measurements, we found that the optimized SU8/Au films (obtained using protocol C) exhibited good stability over twenty eight days of immersion in cell culture medium. In addition, they showed excellent mechanical stability under repeated peel testing with adhesive tape, with the bioelectrode remaining intact over fifty repeat peeling steps. The adhesion and viability of HEK293-T cells on the optimized SU8/Au electrode was found to be comparable to that of HEK293-T cells on glass, confirming the biocompatibility of the electrode.

Finally we note that, although not specifically investigated here, SU8 has previously been used as a photopatternable adhesion layer for multiple substrate materials, including quartz and various plastics, together with a range of conductive materials such as carbon nanotubes, metallic nanoparticles, and conducting polymers such as PEDOT:PSS. Its excellent biocompatibility, combined with the ability to carry out patterning using a simple two-step expose/develop process via a technique known as interlayer lithography^31,66^, suggest SU8-based electrodes will find diverse applications in bioelectronics devices.

## Methods

### Cell cultures

Primary cultures of hippocampal neurons were prepared from embryonic 18-day rat embryos (Charles River). Briefly, hippocampi were dissociated by a 15-min incubation with 0.25% trypsin at 37 °C and cells were plated on poly-L-lysine-coated substrates (0.1% PLL in Borax solution overnight), in Neurobasal supplemented with 2 mM L-glutamine, 2% B27, 100 μg/ml penicillin and 100 μg/ml streptomycin, and with 10% horse serum (Life Technologies) in the first four hours of plating. Biocompatibility data were obtained using two neuronal culture preparations. Human Embryo Kidney (HEK293) cells, obtained from ATCC, were cultured in Dulbecco’s Modified Eagle Medium (DMEM) supplemented with 10% fetal calf serum, 2 mM L-glutamine, 100 μg/ml penicillin, and 100 μg/ml streptomycin (Life Technologies). Cells were plated at an equal density of 10000 cells/cm^2^. Samples were sterilized in 70% ethanol, prior to overnight incubation in 0.1% PLL solution for HEK293 experiments. Cultures were maintained at 37 °C in a humidified atmosphere containing 5% CO_2_.

### Biocompatibility assays

For the fluorescence cytotoxicity assay, attached cells were rinsed twice with imaging solution (in mM: NaCl 140, KCl 2.5, MgCl_2_ 1, CaCl_2_ 1.8, HEPES 20) and were then incubated for 4 min in stain-supplemented imaging solution containing 15 μg/ml of Fluorescein diacetate (FDA; Sigma-Aldrich), 5 μg/ml of Propidium iodide (PI; Sigma-Aldrich) and 3 μg/ml of Hoechst-33342 (Sigma-Aldrich). Cells were washed again twice with imaging solution. Each time point shown in Fig. 1 represents a mean of 1280 ± 116 neurons analyzed per culture substrate condition. For each time point, images were acquired from 3–4 samples per condition by a C91006 CCD Camera (Hamamatsu Photonics Italia) mounted on a Nikon Ti-E epifluorescence microscope (Nikon Instruments). Standard 4′-6-diamidino-2-phenylindole (ex: D350/50×, em: D460/50 m, dic: 400dclp), fluorescein isothiocyanate (ex: D480/30×, em: D535/40 m, dic: 505dclp) and tetramethylrhodamine isothiocyanate (ex: D540/25×, em: D605/55 m, dic: 565dclp) filter sets were used to image Hoechst-33342, fluorescein diacetate and propidium iodine, respectively. Analysis was carried out manually, using the Hoechst signal as the region of interest for each cell. Cells were grouped as follows to generate population percentages: healthy cells were fluorescein positive and PI negative, pyknotic cells were PI positive, and esterase-inactive cells were those with neither a PI nor an FDA signal. HEK staining included FDA and Hoechst dyes, and was carried out on two duplicate samples per condition.

Neurons were patch-clamped using a HEKA EPC10 amplifier and digitizer coupled to a Nikon FN1 upright microscope (Nikon Instruments). Recordings were carried out in current-clamp mode and digitized with a 20 kHz sample rate. The current step protocol was administered after whole cell access, using 500 ms steps of 25 pA increasing from 0 to 375 pA. Standard intracellular (in mM: K-Gluconate 126, NaCl 4, MgSO4 2, CaCl2 0.2, Bapta 0.08, Glucose 9.45, Hepes 5, ATP 3 and GTP 0.1) and extracellular (in mM: NaCl 135, KCl 5.4, MgCl_2_ 1, CaCl_2_ 1.8, HEPES 5, glucose 10 and pH adjusted to 7.4 with NaOH) solutions were used.

### Preparation of SU8-coated glass

Glass substrates were rinsed with deionized (DI) water, acetone and 2-propanol, and then dried. The SU8 films were prepared from the commercial formulation SU8-2 from MicroChem, using the treatment protocols described in the main text. The SU8-2 films were spin-coated onto the cleaned glass at 500 rpm for 6 s and then at 6000 rpm for 30 s. Exposure of the samples was carried out through the glass substrate for a duration of 25 s, using near UV (350–400 nm) illumination at an energy density of 100 mJ/cm^2^.

### Preparation of SU8/Au-coated glass

Glass substrates were cleaned as described above. As received SU8-2 was spin-coated onto the cleaned glass at 500 rpm for 6 s and then at 6000 rpm for 30 s. The SU8 films were coated with 30 nm of gold, using thermal evaporation at a pressure of 10^−6^ mBar. The samples were subjected to various treatment protocols before and after gold deposition as described in the main text. Exposure was carried out through the glass substrate for a duration of 25 s, using near UV (350–400 nm) illumination at an energy density of 100 mJ/cm^2^.

### Transmission spectra

Transmission spectra in the range 400 nm to 1200 nm were measured with a Shimadzu UV-3101 NIR-Vis-NUV spectrophotometer. The samples in PBS and cell culture medium were washed by rapid immersion in DI water prior to measurement.

### Imaging

Optical images in reflection and transmission mode were obtained using an optical microscope (Olympus BX51) fitted with a digital 9 MP camera (Conrad DP-M17. Unpolarised white light illumination was provided by a white light-emitting diode (Thorlabs LIU-PS).

### Measurement of sheet resistance

Sheet resistances were measured at three locations per substrate, using a home-built four-point-probe set-up with equidistant probe spacings of *d* = 1.5 mm. Two Keithley 6514 source-measure units were used to supply a known current *I* between the outer probes, while measuring the voltage *V* across the inner probes. Sheet resistances *R*_s_ were determined according to the thin-film equation:

$${R}_{s}=\frac{\pi }{\mathrm{ln}\,2}(\frac{V}{I})$$

### Peel test procedure

The samples were secured to a wooden plate using double-sided adhesive tape on the underside of the glass substrate. Duct tape (Tesa®) was then placed on the sample and peeled away vertically, using a stepper motor-driven pulley system at a constant velocity of 50 mm/s^67^. The procedure was repeated fifty times or until the film detached from the substrate, whichever occurred first.

### Data statement

The datasets generated and analysed during the current study are available in the Imperial College Box repository at https://imperialcollegelondon.box.com/v/su8gold.

## Electronic supplementary material

Supplementary Information
