# Supplementary material for: Use of SU8 as a stable and biocompatible adhesion layer for gold bioelectrodes
Source: Sci Rep. 2018 Apr 3;8:5560. doi: 10.1038/s41598-018-21755-6 (PMC5882823; doi:10.1038/s41598-018-21755-6)
Supplement: Supplementary file 1 — Supplementary Information [file 41598_2018_21755_MOESM1_ESM.pdf]

Use of SU8 as a stable and biocompatible adhesion layer for gold bioelectrodes

Bruno F.E. Matarèse[1], Paul L.C. Feyen[2], Aniello Falco[3], Fabio Benfenati[2,4], Paolo Lugli[3], John C. de Mello[1]

[1]Imperial College London, Exhibition Road, South Kensington, London SW7 2AY, UK
[2]Center for Synaptic Neuroscience and Technology, Istituto Italiano di Tecnologia, 16132 Genoa, Italy
[3]Faculty of Science and Technology, Free University of Bolzano, Bozen, 39100 Bolzano, Italy
[2,4]Department of Experimental Medicine, University of Genoa, 16132 Genoa, Italy

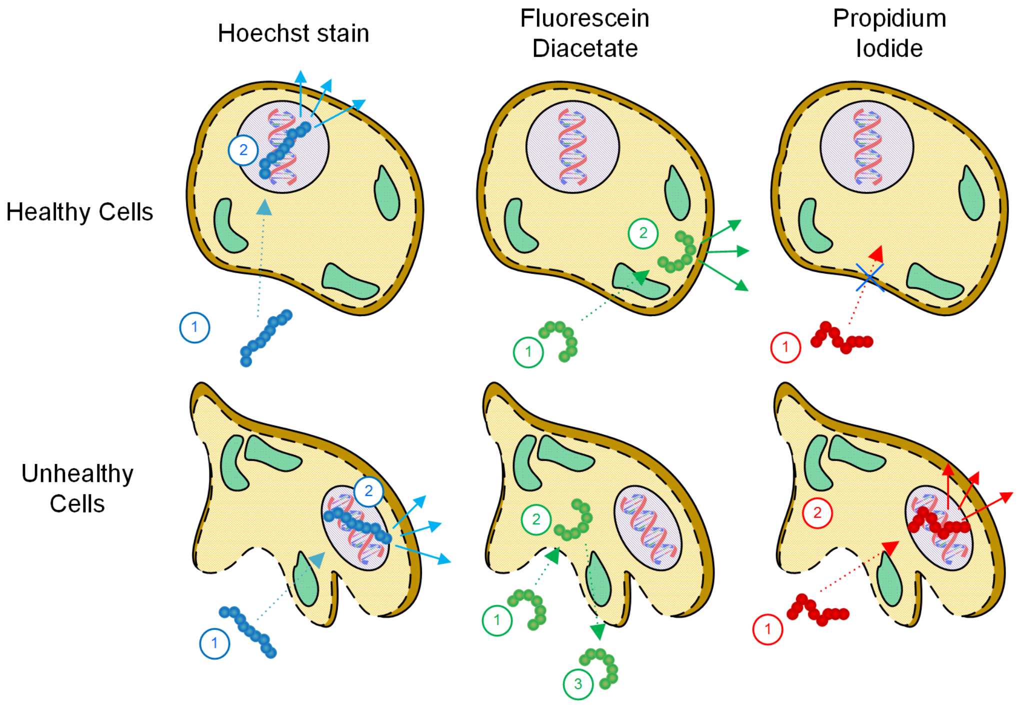

Fig. S1 Schematic illustrating the interaction of Hoechst stain, fluorescein diacetate (FDA) and propidium iodide (PI) with healthy and unhealthy cells. Hoechst penetrates the nuclei of healthy and unhealthy cells, providing a visual indicator of general cell adhesion (whether healthy or not). FDA exhibits fluorescence only when present in healthy cells, while PI is only able to penetrate the membranes of pyknotic cells. Healthy cells exhibit an FDA signal but no PI signal; pyknotic cells exhibit only a PI signal; while esterase-inactive cells exhibit neither a PI signal nor an FDA signal.
